# "Normal but Catastrophic” Euglycemic Diabetic Ketoacidosis Precipitated by Sodium-Glucose Cotransporter-2 Inhibitor Use: A Case Report

**DOI:** 10.7759/cureus.49236

**Published:** 2023-11-22

**Authors:** Myrem Jesame F Sarno, Dan Philip F Hernandez, Melgar O Matulac

**Affiliations:** 1 Internal Medicine, Adventist Medical Center Manila, Pasay City, PHL; 2 Endocrinology, Diabetes and Metabolism, Adventist Medical Center Manila, Pasay City, PHL; 3 Interventional Cardiology, Adventist Medical Center Manila, Pasay City, PHL

**Keywords:** diabetic ketoacidosis and sglt2 inhibitor use, diabettic ketoacidosis and sglt2 inhibitor use, dka and trauma, euglycemic diabetoketoacidosis, diabetic ketoacidosis (dka), euglycemic dka

## Abstract

Euglycemic diabetic ketoacidosis (EuDKA) is an uncommon diabetic complication. Just like diabetic ketoacidosis (DKA), EuDKA is a medical emergency. EuDKA is primarily related to the imbalance between insulin and counter-regulatory hormones, with an elevated insulin/glucagon ratio, and is characterized by blood glucose near normal (blood glucose less than 250 mg/dL) in the background of DKA. There are many factors associated with EuDKA, but the overall mechanism is based on a relative state of carbohydrate deficit, resulting in ketosis while maintaining near-normal glucose levels. Sodium-glucose cotransporter 2 (SGLT2) inhibitors are a new oral antidiabetic medication category that can precipitate EuDKA. EuDKA is more common in patients with diabetes mellitus on SGLT2 inhibitors with lower mass index and decreased glycogen store which can be triggered by surgery, infection, trauma, a major illness or reduced food intake and persistent vomiting, gastroparesis, dehydration, and reduced insulin dosages.

This is a case of a 34-year-old male, Filipino, diagnosed with type 2 diabetes mellitus, who was maintained on dapagliflozin + metformin 5mg/1000mg taken twice a day with good compliance and was admitted with EuDKA precipitated by decreased food intake and managed with intravenous insulin. Throughout admission, the blood glucose levels did not exceed 250mg/dL. His clinical condition improved through insulin therapy, administration of sodium bicarbonate, and intravenous hydration.

EuDKA is an uncommon diabetic complication. High clinical suspicion is required to avoid delay in diagnosis and management since normal blood sugar levels masquerade the underlying DKA. Nevertheless, the cornerstone for the management of DKA and EuDKA remains the same: intravenous hydration and insulin therapy.

## Introduction

Diabetic ketoacidosis (DKA), a serious complication of diabetes that can lead to diabetic coma and even death, is a clinical syndrome that can occur in both type 1 and type 2 diabetes. Based on the available data and studies for DKA, it is shown that mortality rates are at <1% to 1.8% in the United Kingdom, 0.65% to 3.3% in Canada, and as high as 30% in countries with “less privileged health system” [1]. It is a medical emergency that usually presents with hyperglycemia (glucose of >250mg/dL), ketosis, and high anion gap metabolic acidosis [2-4]. Euglycemic diabetic ketoacidosis (EuDKA) is a relatively new phenomenon that has most commonly been characterized by near-normal glucose levels (<250 mg/dL) in the presence of severe metabolic acidosis and ketosis [4,5]. 

Sodium-glucose cotransporter-2 inhibitors (SGLT2i), a novel class of antidiabetic drugs that block the reabsorption of filtered glucose from the proximal convoluted tubule thereby enhancing its excretion, are associated with EuDKA [6]. In type 1 diabetes mellitus, lipolysis and ketogenesis result from insulin reduction during SGLT2i initiation. In type 2 diabetes mellitus, glucosuria leads to increased preproglucagon gene expression leading to a rise in pancreatic α‐cell glucagon levels. The increased glucagon/insulin ratio stimulates lipolysis and hepatic ketogenesis. In addition, SGLT2i may also decrease renal clearance of ketones hence leading to metabolic acidosis [6].

Based on available data, about 2.6% to 3.2% of DKA admissions are euglycemic [7]. The incidence of SGLT2i-associated DKA in T1DM and T2DM is 6% and 0.1-0.6% respectively [6]. In a multicenter study, the incidence of EuDKA among SGLT2i users, including Southeast Asians and Filipinos, is 0.25% [6]. Currently, there are no available local data on the incidence of EuDKA in the Philippines.

SGLT2 inhibitors produce EuDKA following the loss of urinary glucose creating a state of carbohydrate deficit and volume depletion, increasing the glucagon/insulin ratio, resulting in a state of severe dehydration and ketosis [3,7]. Precipitating events include infection, post-trauma, dehydration, nausea, vomiting, and diarrhea [3,6].

Since normal blood sugar levels masquerade the underlying DKA, high clinical suspicion and prompt recognition of risk factors would help clinicians avoid the delay in diagnosis and management.

Herein, we present a case of a young Filipino adult with T2DM who is on an SGLT2 inhibitor with signs and symptoms of DKA but with near-normal glucose levels.

## Case presentation

A 34- year-old male, diagnosed with T2DM four years before, presented to the emergency room with epigastric pain, nausea, vomiting, generalized body weakness, and shortness of breath. 

The patient had a history of a motor vehicular accident sustaining a hematoma on the right periorbital area four days prior to consultation. A plain cranial CT scan was initially done which showed unremarkable results and he was sent home with analgesia (Celecoxib 200 mg tab as needed for pain). 

Two days prior to the consult, he started to experience dizziness, nausea, vomiting, and loss of appetite. A repeat cranial CT scan was done and revealed an unremarkable result. The patient was given vestibular suppressant and antiemetic drugs. There was no associated fever, chills, sore throat, cough, chest pain, flank pain, or dysuria, and the patient’s symptoms progressed with weakness, epigastric pain radiating to the back, nausea, and vomiting which prompted ER consult and subsequent admission. 

The patient is a public school teacher with no history of recent travel, intake of unsanitary food, or known sick contact. He was a known diabetic (Type 2) for four years and was maintained on dapagliflozin + metformin 5mg/1000mg/tab twice daily wherein he claimed to have fair compliance, no hospital visit, nor consultation done for the past three years. He has no CBG monitoring at home and cannot recall his last HbA1c and FBS results. He was diagnosed with congenital nystagmus but is controlled with prescription glasses. There were no known allergies to food and medications and no alcohol intake, smoking, or use of illicit drugs. 

The patient came in normotensive (BP of 130/80 mmHg), afebrile (temperature of 36.5), tachycardic (HR of 130 bpm), and tachypneic (RR of 30-36 cpm). He has a BMI of 23kg/m^2^. He was weak-looking and drowsy but responded upon verbal stimulation. On examination, he had a hematoma on the right periorbital area with conjunctival hemorrhage and dry oral mucosa. Heart sounds were distinct with no murmur. The abdomen was soft but with direct tenderness on the epigastric area, no splenomegaly nor hepatomegaly, with bounding pulses and no edema. 

Spot capillary blood glucose was 230 mg/dL. Arterial blood gas analysis showed partially compensated metabolic acidosis with adequate oxygenation (Table 1) [7]. Urinalysis showed glucose and ketonuria but no infection. HbA1c was elevated at 14%, with multiple electrolyte imbalances: hyponatremia, and hyperkalemia. The anion gap was high with elevated serum osmolality. Complete blood count showed hemoconcentration that may be due to severe dehydration (hemoglobin 177, hematocrit 0.54) platelet 382, and leukocytosis (WBC 18.90) with a predominance of PMNs (87%). Transaminases (SGPT 10, SGOT 20), pancreatic enzymes (lipase 93, amylase 10.5), and creatinine were normal. Infection was ruled out when chest x-ray, urinalysis, procalcitonin, and whole abdominal ultrasound yielded normal results, and repeat CBC after hydration showed resolution of leukocytosis even without antibiotic use. 12L-ECG showed sinus tachycardia without ischemic changes or chamber enlargements. Differential diagnoses upon initial evaluation were acid peptic disease and acute pancreatitis which were eventually ruled out.

**Table 1:** Laboratory Values of Diabetic Ketoacidosis and Euglycemic DKA (Representative Ranges at Presentation) in Comparison to the Patient's Results DKA: Diabetic ketoacidosis; EuDKA: euglycemic ketoacidosis

Parameters	DKA	EuDKA	Patient's Values
Glucose (mg/dL)	250-600	<200-250	230
Sodium (mmoL/L)	125-135	~135	134
Potassium (mmoL/L)	Normal to increase	Normal to increase	5.2
Magnesium (mmoL/L)	Normal	Normal	0.87
Chloride (mmoL/L)	Normal	Normal	97.7
Creatinine (umoL/L)	Slightly to moderately increased	Slightly increased	74
Serum Osmolality (mOsm/kg)	300-340	~300	308.4 H
Urine Ketones (dipstick)	++++	++++	++++
Serum bicarbonate (mEq/L)	<18	<18	1.88
Arterial pH	6.9-7.3	6.8-7.3	6.79
Arterial pCO_2_ (%)	20-30	20-30	8.3
Anion gap (mEq/L)	Increase	Increase	35.34 H

The patient was resuscitated initially with a 1.3L bolus of plain lactated Ringer’s over the first 1-3 hours which was then regulated to 250cc/hr and was titrated accordingly thereafter. The patient was immediately started on insulin therapy i.e. intravenous regular insulin and subcutaneous long-acting insulin (Glargine 15 units) and was given NaHCO3 50 mEqs IV bolus. Oral anti-diabetic drugs were put on hold as well. The patient was also referred to toxicology service due to consideration of possible drug-drug interaction reaction, and nephrology service since the patient had persistent metabolic acidosis on the second hospital day. He was given NaHCO3 100mEqs via a drip for two cycles with a noted resolution of acidosis and symptoms thereafter. Throughout hospitalization, the patient’s blood glucose levels range from 98-201 mg/dL which is within the glucose goal of 150-200mg/dL or 8.3-11.1 mmol/L.

The patient’s condition and well-being improved and he was discharged on the fourth hospital day. Acidosis was resolved, and glucose trends were maintained on normoglycemic levels. He was given subcutaneous insulin glargine 18 units once daily and sitagliptin + metformin 50/500mg tab twice daily as his maintenance medications upon discharge. The patient had regular visits at the outpatient department wherein subcutaneous insulin was tapered and eventually discontinued because of improving glucose and HBA1C control.

## Discussion

EuDKA is an uncommon form of DKA with a near-normal glucose concentration (in contrast to classic DKA with glucose >250mg/dL) involving a general state of carbohydrate deficit and volume depletion resulting in ketosis [8-10]. 

The underlying mechanism of EuDKA is secondary to a carbohydrate deficit resulting in generalized decreased serum insulin and excess counter-regulatory hormones like glucagon, epinephrine, and cortisol. The increased glucagon/insulin ratio leads to increased lipolysis, increased free fatty acids, and ketoacidosis. Production of ketone bodies (beta-hydroxybutyrate, acetoacetate, and acetone) in EuDKA is similar to DKA and is responsible for metabolic acidosis [3]. The resulting anion gap metabolic acidosis triggers respiratory compensation and the sensation of dyspnea, as well as nausea, anorexia, and vomiting. Volume depletion and dehydration resulting from decreased oral intake, vomiting, and osmotic diuresis from glucosuria, further exacerbate elevations in glucagon, cortisol, and epinephrine, worsening lipolysis and ketogenesis. Additionally, decreased gluconeogenesis by the liver occurs in fasting where hepatic glycogen is already depleted, or increased glucosuria by the kidneys contributes to EDKA [3,5,8,9].

The emerging use of SGLT2 inhibitors (dapagliflozin, empagliflozin, and canagliflozin) is associated with an increased risk of developing EuDKA. The exact incidence rate of SGLT2 inhibitors associated with DKA is still unknown [9].

The exact mechanism through which SGLT2 inhibitors can lead to euDKA is not fully understood, but several theories exist.

Increased ketone production

SGLT2 inhibitors lower blood glucose levels by increasing urinary glucose excretion. This may lead to a shift in the body's metabolism, prompting an increase in the production of ketones from fats as an alternative energy source. If insulin levels are insufficient to regulate this increased ketone production, it can lead to ketoacidosis despite normal or slightly elevated blood glucose levels [11,12].

Glucose-independent ketone formation 

SGLT2 inhibitors might stimulate glucagon secretion and decrease insulin secretion independently of glucose levels. This alteration in hormonal balance could promote increased ketone production [13]. 

Dehydration and volume depletion

SGLT2 inhibitors can cause increased urination (due to increased glucose excretion). This may lead to dehydration and volume depletion, contributing to the risk of ketoacidosis [13,14].

Reduced insulin secretion

Some studies suggest that SGLT2 inhibitors might reduce insulin secretion by affecting pancreatic beta cells, potentially contributing to ketoacidosis [15,16].

In the case of our patient, identified risk factors for developing EuDKA include intake of SGLT2i which was precipitated by the decreased dietary intake post-trauma. 

While the management of EuDKA is similar to DKA, an immediate and prompt diagnosis should be made to avoid delay in the management and treatment. Adequate fluid resuscitation and insulin administration should be immediately started. End organ damage should also be investigated to avoid further complications [5,6]. Case reports describe EuDKA’s longer resolution of up to 92 hours compared to hyperglycemic DKA of 35 hours due to prolonged acidosis [6,10,11].

At present, there are no data or reports regarding the incidence and prevalence of EuDKA in the Philippines given the limited cases and its rarity [6].

## Conclusions

Absolutely, the occurrence of EuDKA precipitated by SGLT2 inhibitors is considered rare but recognized within the medical literature. However, due to its infrequent incidence and the unique nature of this form of ketoacidosis (with near-normal blood glucose levels), it warrants a higher index of suspicion among healthcare providers. Continued research into the precise mechanisms by which SGLT2 inhibitors may contribute to EuDKA is necessary for a comprehensive understanding and better management strategies. It's crucial for healthcare professionals to be vigilant and recognize the potential risk factors and symptoms associated with EuDKA in patients using SGLT2 inhibitors.

Patients prescribed these medications should receive thorough education about the signs and symptoms of ketoacidosis, emphasizing the importance of seeking immediate medical attention if such symptoms arise, even if their blood glucose levels appear within the normal range. Given the rarity of EuDKA as a complication of SGLT2 inhibitors and its potentially severe consequences, a multidisciplinary approach involving ongoing research, vigilant monitoring, and heightened awareness among both healthcare providers and patients is crucial for early detection, prompt management, and prevention of complications.
